# MSU-Net: Multi-Scale U-Net for 2D Medical Image Segmentation

**DOI:** 10.3389/fgene.2021.639930

**Published:** 2021-02-11

**Authors:** Run Su, Deyun Zhang, Jinhuai Liu, Chuandong Cheng

**Affiliations:** ^1^Institute of Intelligent Machines, Hefei Institutes of Physical Science, Chinese Academy of Sciences, Hefei, China; ^2^Science Island Branch of Graduate School, University of Science and Technology of China, Hefei, China; ^3^School of Engineering, Anhui Agricultural University, Hefei, China; ^4^Department of Neurosurgery, The First Affiliated Hospital of University of Science and Technology of China (USTC), Hefei, China; ^5^Division of Life Sciences and Medicine, University of Science and Technology of China, Hefei, China; ^6^Anhui Province Key Laboratory of Brain Function and Brain Disease, Hefei, China

**Keywords:** multi-scale block, U-net, medical image segmentation, convolution kernel, receptive field

## Abstract

Aiming at the limitation of the convolution kernel with a fixed receptive field and unknown prior to optimal network width in U-Net, multi-scale U-Net (MSU-Net) is proposed by us for medical image segmentation. First, multiple convolution sequence is used to extract more semantic features from the images. Second, the convolution kernel with different receptive fields is used to make features more diverse. The problem of unknown network width is alleviated by efficient integration of convolution kernel with different receptive fields. In addition, the multi-scale block is extended to other variants of the original U-Net to verify its universality. Five different medical image segmentation datasets are used to evaluate MSU-Net. A variety of imaging modalities are included in these datasets, such as electron microscopy, dermoscope, ultrasound, etc. Intersection over Union (IoU) of MSU-Net on each dataset are 0.771, 0.867, 0.708, 0.900, and 0.702, respectively. Experimental results show that MSU-Net achieves the best performance on different datasets. Our implementation is available at https://github.com/CN-zdy/MSU_Net.

## 1. Introduction

Medical imaging analysis has made a significant breakthrough with the rapid progress of deep learning (Long et al., 2015; Chen et al., 2018a; Salehi et al., 2018; Wang et al., 2019b). Among these techniques, encoder-decoder architecture has been widely used in the medical image segmentation task (Salehi et al., 2017; Xiao et al., 2018; Guan et al., 2019). U-Net (Ronneberger et al., 2015) is the most classic encoder-decoder structure for medical image segmentation. In recent years, the original U-Net has been modified by many researchers. As a result, many variants of the original U-Net have been proposed (Poudel et al., 2016; Oktay et al., 2018; Roth et al., 2018).

However, the variants of the original U-Net come with two limitations. First, the diversity of features is lost due to the fixed receptive field of the convolution kernel. The same scale feature maps extracted from the convolution kernel with different receptive fields are semantically different. As a result, the performance of the network may vary with the size of the receptive field, and the performance depends on the size of the receptive field in the convolution kernel. Redundant features will be extracted when the receptive field of the convolution kernel is too small. Smaller targets are ignored when the receptive field of the convolution kernel is too large. For example, in the pulmonary lesion or multi-organ segmentation task, the edge detail of the smaller lesion/organ is not fine by the large receptor field and the structure of the lesion/organ is not obvious by the small receptor field. Therefore, it is very important to use the convolution kernel with different receptive fields to process the image (Luo et al., 2016; Peng et al., 2017; Shen et al., 2019). In the natural image processing task, satisfactory results are obtained by combining the convolution of different receptive fields (Seif and Androutsos, 2018). To the best of our knowledge, there are few reports based on different receptive fields in medical image segmentation tasks. Second, some information may be lost using a single convolutional sequence to extract features at each scale. More feature information can be obtained by multiple convolutional sequences. The loss of feature information can be reduced by the structure of multiple convolutional sequences in the process of down-sampling and up-sampling. Therefore, the learning capacity of the network is aided by multiple convolutional sequences (He et al., 2015).

In this paper, a new image segmentation architecture (multi-scale U-Net) is proposed by us to overcome the above limitations. This architecture is a generalization segmentation architecture. Multi-scale U-Net (MSU-Net) consists of blocks of multi-scale whose multi-scale blocks are composed of convolution sequences with different receptive fields. The multi-scale block introduced in MSU-Net achieves the following advantages. First, more feature information can be obtained because of the multiple convolutional sequences structure embedded in the network. The input of the convolution sequence is all the same, while their convolution kernel is not shared. This design not only improves the performance of segmentation but also facilitates the learning of network in the training process. Second, the features extracted from the multi-scale block are diversified. This is caused by the multiple convolution sequences with different receptive fields in multi-scale block. This is helpful for intensive forecasting tasks that require detailed spatial information. The semantics extracted from the convolution sequence with different receptive fields are different on the same scale feature map. This structure enables the encoder of the network to extract features better and the decoder to restore features better. We construct different types of multi-scale blocks with several commonly used convolution kernels. An extensive evaluation of different types of multi-scale blocks is performed on three segmentation datasets. Our results demonstrate that MSU-Net built by integrated multiple convolution sequences with different receptive fields enables significant improvement of semantic segmentation. Compared with the traditional U-Net architecture, the main improvement of MSU-Net is the integration of multiple convolution sequences with different sizes of receptive fields. This improvement enables the object features to become more conspicuous with forward propagation. In addition, the proposed multi-scale block can be easily integrated into other network structures.

In summary, the main contributions of this paper are summarized as follows:

(1) Multi-scale blocks are proposed by us based on several commonly used convolution kernel. More diverse feature information and better feature maps are captured from the images through multi-scale block.

(2) MSU-Net, a new segmentation architecture for medical image, is proposed for medical image segmentation. This is an improvement on the basic structure of U-Net. Compared to the existing algorithms, the proposed method has a stronger ability to overcome the problems of class-imbalance and overwhelmed.

(3) Different receptive fields are crucial for dense prediction tasks requiring detailed spatial information. It can stimulates learning capacity of network and make the network more robust. Experimental results demonstrate that the proposed method is outperforms the state-of-the-art methods in medical image segmentation task under different imaging modalities.

## 2. Related Works

With the development of convolutional neural network (CNN) in the field of natural image processing and medical image analysis, automatic feature learning algorithm using deep learning has become a feasible method for biomedical image segmentation (Le et al., 2019, 2020; Sua et al., 2020). Segmentation method based on deep learning is a learning method with pixel-classification, which is different from the traditional pixel or superpixel classification method (Abramoff et al., 2007; Kitrungrotsakul et al., 2015; Tian et al., 2015) using hand-made features. The limitations of hand-made features are overcome when deep learning approaches are used to learn features. The limitations of hand-made features are overcome when deep learning approaches are used to learn features. Early deep learning methods for medical image segmentation are mostly based on patch. The strategy based on plaque and sliding window was proposed by Ciresan et al. (2012) to segment neuronal membranes from microscopic images. Kamnitsas et al. (2017) adopted a multi-scale 3D CNN architecture with fully connected conditional random field (CRF) to enhance patch based brain leasion segmentation. Pereira et al. (2016) proposed an automatic segmentation method based on CNN to segment brain tumors. Obviously, two main drawbacks are introduced by this solution: the redundant computation caused by sliding window and the global feature cannot be learned.

With the emerging of end-to-end FCN (Long et al., 2015), Ronneberger et al. (2015) proposed U-Net for biomedical image segmentation. U-Net has shown good performance in fields of medical image segmentation. It has become a popular neural network architecture for biomedical image segmentation tasks (LaLonde and Bagci, 2018; Fan et al., 2019; Song et al., 2019). Li et al. (2019) proposed a new dual-U-Net architecture to solve the problem of nuclei segmentation. Milletari et al. (2016) proposed a 3D image segmentation method based on U-Net to perform end-to-end training on prostate MRI. Guan et al. (2019) proposed an improved CNN structure for removing artifact from 2D PAT images reconstructed. Many variants of U-Net has been appeared for different medical image segmentation tasks. In order to improve the learning ability of feature, some new modules are proposed to replace the original modules. Seo et al. (2019) proposed an up-sampling method based on an object and redesigned the remaining paths and skip-connection. The limitation of the traditional U-Net algorithm was overcome in this way. Ge et al. (2019) proposed a k-shaped network of end-to-end deep neural network. The network was used for multi-view segmentation and multi-dimensional quantification of LV in PEAV sequences. Myronenko (2018) proposed a semantic segmentation method for 3D brain tumor segmentation from multimodal 3D MRIs. An asymmetric encoder was used to extract features, and then two decoders segment the brain tumor and reconstruct the input image, respectively. Oktay et al. (2018) proposed AttU-Net in combination with attention gate. Alom et al. (2018) integrated the structure of Recurrent Neural Network (RNN) and ResNet into the original U-Net. RNN could make the network extract better features. ResNet enables the training of deeper networks. Liu et al. (2020) proposed a ψ-shaped depth neural network (ψ-Net). In the deep stage, semantic information was featured by selective aggregation. In the shallow stage, the semantic information obtained in the deep stage was used to improve the detailed information. Therefore, discriminative features were obtained to provide the basis for accurate subcortical segmentation of brain structures. In addition to the above achievements in medical image segmentation based on U-Net, some researchers have also improved U-Net to apply in general image segmentation. Zhang et al. (2018) proposed a semantic segmentation neural network based on residual learning and U-Net for road area extraction. Kohl et al. (2018) proposed a generative segmentation model based on a combination of a U-Net with a conditional variational auto-encoder. A new Recurrent U-Net had been proposed by Wang et al. (2019a). This model not only retained the compactness of U-Net, but also achieved a good performance improvement in some benchmarks. TernausNet was proposed by Iglovikov and Shvets (2018). The network replaces the encoder in U-Net with VGG11 and conducts pre-training on ImageNet. TernausNet achieved the best results in the Kaggle Carvana Image Masking Challenge.

Although the architecture of U-Net has been widely used, the most basic architecture has not changed. The convolution blocks of the original U-Net network are adjusted by us to improve the efficiency of the segmentation algorithm. The convolution blocks are arranged in parallel to form a multiple convolution sequence. Richer semantic information is provided by this design. In addition, the convolution kernel of the multiple convolution sequence is adjusted to have different receptive fields. The convolution kernel with different receptive fields enables the network to better extract and restore features.

## 3. Method

**Figure 1:**
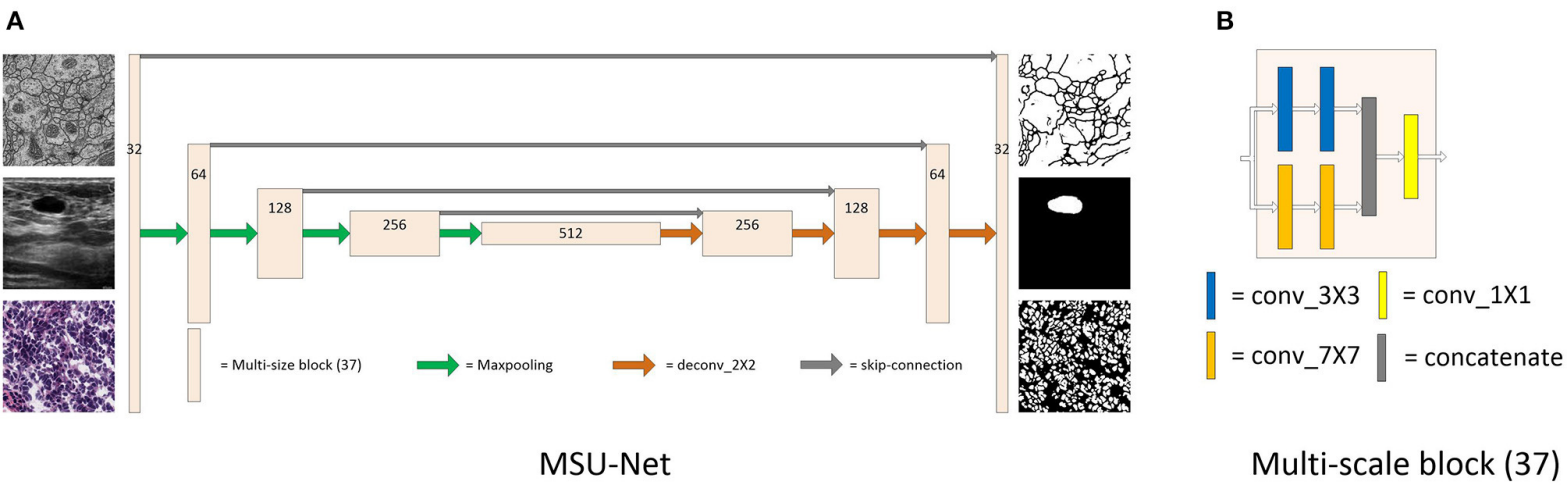
Detailed description of MSU-Net and multi-scale block (37). Panel **(A)** is the architecture of MSU-Net. The overall architecture is similar to the original U-Net. The dimensions of the network are represented by numbers on the block structure. Panel **(B)** is the architecture of multi-scale block (37). This module is embedded in the original U-Net to get MSU-Net.

The proposed MSU-Net consists of major part: multi-scale block (37), as shown in Figure 1. In the following, we first trace the types of multi-scale block and then explain the structure of MSU-Net and extended work of multi-scale block.

### 3.1. Multi-Scale Block

**Figure 2:**
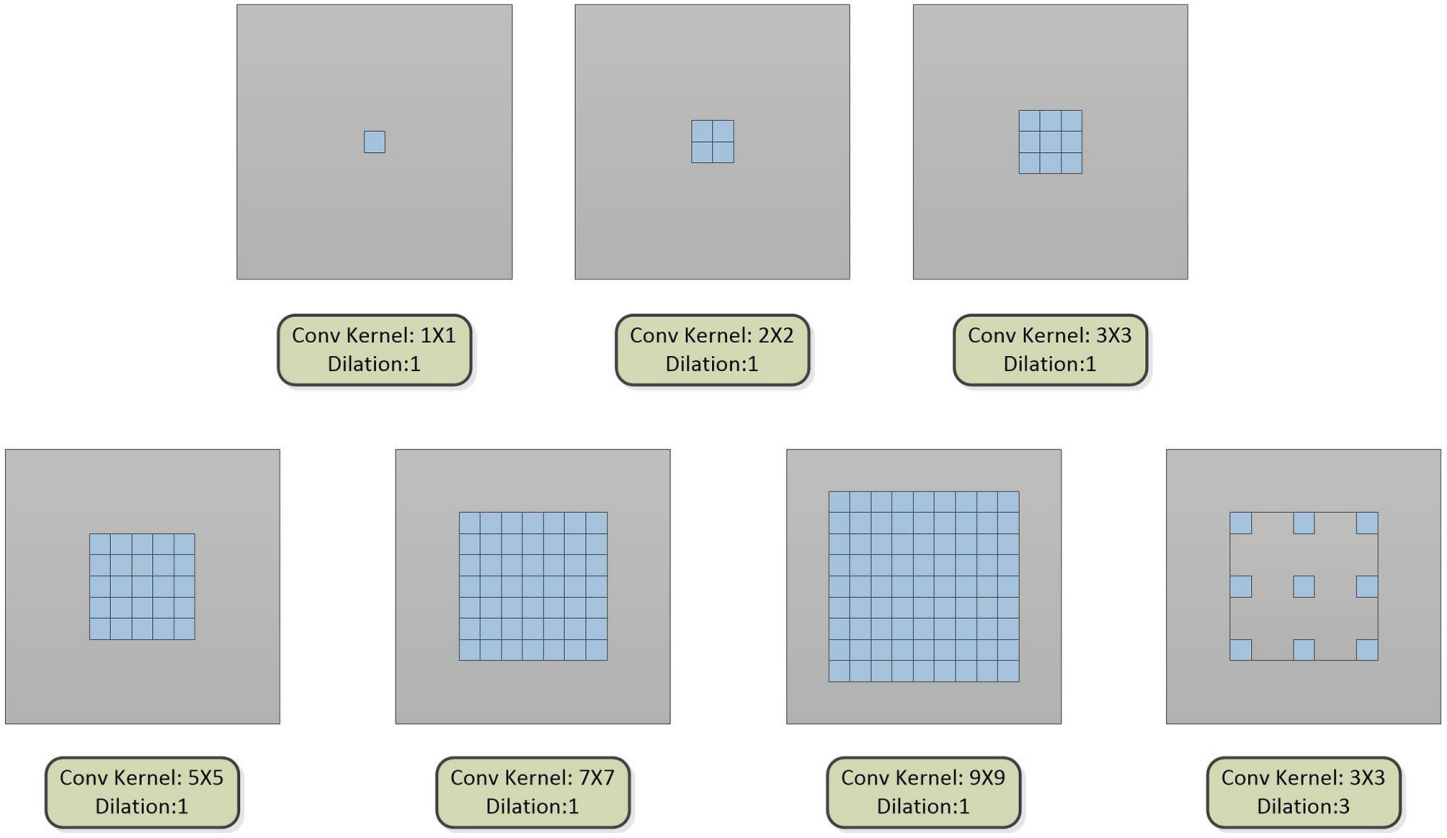
The type of convolution kernel used in this article. Combining the above seven convolution kernels, different types of multi-scale blocks are proposed.

The multi-scale block is proposed by us, which is composed of multiple convolution sequences with different receptive fields. More diverse semantic information is extracted by this module and more detailed feature maps are generated. The widely used convolution kernel is shown in Figure 2.

**Figure 3:**
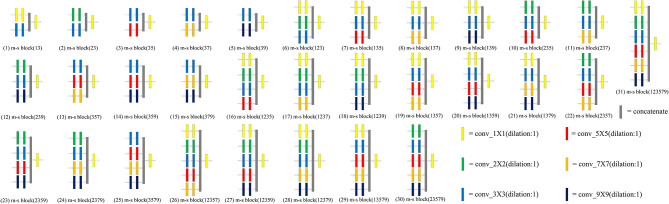
An overview of 31 multi-scale blocks. m-s block represents the multi-scale block. Different multi-scale blocks are designed according to several commonly used convolution kernels. More richer and diverse features can be extracted through this design. Meanwhile, the problem of unknown network width can be alleviated by this design. Conducive to intensive prediction tasks that require detailed spatial information.

The convolution kernel with different receptive fields is matched to obtain a multi-scale block. We designed 31 kinds of multi-scale blocks according to the above several convolution kernels. The multi-scale block evolved from the different convolution kernels is shown in Figure 3.

**Figure 4:**
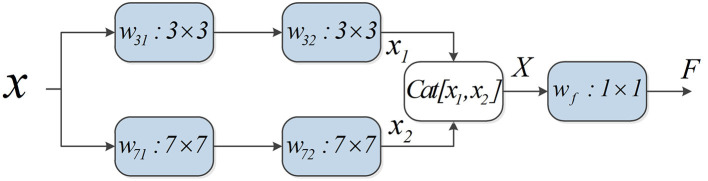
Detailed description of multi-scale block. First, two 3X3 and 7X7 convolution kernels are used to extract features. Second, the extracted features are merged by the feature by cat. Finally, the fused features are output after dimensionality reduction by 1X1 convolution.

The 3 × 3 convolution kernel has been used in all experiments. The features of the input multi-scale block are processed by the convolution kernel with different receptive fields, and then the obtained features are output after 1 × 1 convolution. A comprehensive ablation experiment is used to verify the performance of different types of multi-scale blocks. In the experiment, three datasets are used by us. The datasets are EM, BUL, and CXR, respectively (detailed in section 4.1). The experiments are carried out after integrated each multi-scale block into the original U-Net. The experimental results are illustrated in Table 1. The performance of multi-scale block (37) is the best. The details of multi-scale block (37) are shown in Figure 4.

**Table 1:** Ablation study on MSU-Nets of the convolution kernel with different receptive fields.

**Applications**	**BUL**	**EM**	**NS**
	**M ± SD**	**M ± SD**	**M ± SD**
MSU-Net (13)	0.548 ± 0.076	0.871 ± 0.002	0.678 ± 0.017
MSU-Net (23)	0.610 ± 0.029	0.840 ± 0.035	0.661 ± 0.028
MSU-Net (35)	0.690 ± 0.047	0.884 ± 0.017	0.670 ± 0.036
MSU-Net (37)	**0.708** **±** **0.011**	**0.900** **±** **0.001**	**0.702** **±** **0.010**
MSU-Net (39)	0.699 ± 0.016	0.895 ± 0.009	0.660 ± 0.011
MSU-Net (123)	0.547 ± 0.067	0.862 ± 0.012	0.672 ± 0.015
MSU-Net (135)	0.679 ± 0.005	0.883 ± 0.010	0.676 ± 0.021
MSU-Net (137)	0.696 ± 0.018	0.890 ± 0.015	0.684 ± 0.025
MSU-Net (139)	0.682 ± 0.037	0.880 ± 0.015	0.674 ± 0.020
MSU-Net (235)	0.673 ± 0.036	0.873 ± 0.023	0.684 ± 0.025
MSU-Net (237)	0.703 ± 0.042	0.888 ± 0.017	0.687 ± 0.019
MSU-Net (239)	0.664 ± 0.029	0.893 ± 0.011	0.672 ± 0.023
MSU-Net (357)	0.679 ± 0.018	0.888 ± 0.016	0.682 ± 0.015
MSU-Net (359)	0.693 ± 0.007	0.894 ± 0.006	0.686 ± 0.020
MSU-Net (379)	0.705 ± 0.008	0.894 ± 0.011	0.671 ± 0.023
MSU-Net (1,235)	0.652 ± 0.015	0.877 ± 0.015	0.662 ± 0.038
MSU-Net (1,237)	0.655 ± 0.008	0.886 ± 0.009	0.693 ± 0.025
MSU-Net (1,239)	0.699 ± 0.017	0.885 ± 0.014	0.687 ± 0.031
MSU-Net (1,357)	0.689 ± 0.033	0.895 ± 0.005	0.673 ± 0.023
MSU-Net (1,359)	0.700 ± 0.028	0.898 ± 0.002	0.689 ± 0.015
MSU-Net (1,379)	0.702 ± 0.025	0.898 ± 0.003	0.692 ± 0.017
MSU-Net (2,357)	0.694 ± 0.040	0.894 ± 0.004	0.687 ± 0.023
MSU-Net (2,359)	0.681 ± 0.023	0.884 ± 0.014	0.702 ± 0.018
MSU-Net (2,379)	0.694 ± 0.036	0.882 ± 0.014	0.675 ± 0.013
MSU-Net (3,579)	0.696 ± 0.338	0.893 ± 0.010	0.695 ± 0.011
MSU-Net (12,357)	0.680 ± 0.017	0.893 ± 0.005	0.696 ± 0.027
MSU-Net (12,359)	0.705 ± 0.014	0.892 ± 0.006	0.687 ± 0.040
MSU-Net (12,379)	0.667 ± 0.023	0.893 ± 0.002	0.695 ± 0.021
MSU-Net (13,579)	0.697 ± 0.032	0.899 ± 0.001	0.685 ± 0.025
MSU-Net (23,579)	0.705 ± 0.020	0.889 ± 0.014	0.697 ± 0.008
MSU-Net (123,579)	0.693 ± 0.028	0.896 ± 0.002	0.696 ± 0.017

*The numbers in brackets represent the size of receptive field in MSU-Net. This is corresponds to the different multi-scale blocks in Figure 3. Intersection over Union (IoU) is used as the evaluation metric for comparative. Bold values represent the best results*.

*x* represents the characteristics of the input. *x*_1_ and *x*_2_ represent the characteristics obtained by the convolution kernel of different sizes. *F* is the output result of multi-scale block. *F* is computed as follows:

(1) $$\begin{array}{l} x_{1}=w_{32}\left(w_{31}x+b_{31}\right)+b_{32} \end{array}$$

(2) $$\begin{array}{l} x_{2}=w_{72}\left(w_{71}x+b_{71}\right)+b_{72} \end{array}$$

(3) $$\begin{array}{l} X=Cat\left[x_{1},x_{2}\right] \end{array}$$

(4) $$\begin{array}{l} F=w_{f}X+b_{f} \end{array}$$

Feature fusion needs to be used in multi-scale block before 1X1 convolution. Therefore, different fusion methods are validated by us (results in Table 2). MSU-Net (37+sum) uses element summation for feature fusion. MSU-Net (37) uses concatenation for feature fusion.

**Table 2:** Ablation study for MSU-Net and its variants.

**Architecture**	**BUL**	**EM**	**NS**
	**M ± SD**	**M ± SD**	**M ± SD**
MSU-Net	**0.708** **±** **0.011**	**0.900** **±** **0.001**	**0.702** **±** **0.010**
MSU-Net(37+sum)	0.694 ± 0.020	0.894 ± 0.013	0.683 ± 0.017
MSU-Net(encoder)	0.646 ± 0.061	0.889 ± 0.013	0.679 ± 0.021
MSU-Net(decoder)	0.656 ± 0.027	0.883 ± 0.018	0.661 ± 0.024
MSU-Net(37+concatenated)	0.642 ± 0.036	0.899 ± 0.004	0.674 ± 0.024
MSU-Net(73+concatenated)	0.707 ± 0.061	**0.900** **±** **0.001**	0.667 ± 0.022
MSU-Net(37+dilated)	0.640 ± 0.033	0.877 ± 0.005	0.662 ± 0.013

*MSU-Net is MSU-Net (37) in Table 1. MSU-Net (37+ sum) is an MSU-Net with feature fusion by adding. MSU-Net (encoder) and MSU-Net (decoder) are obtained by using multi-scale block to replace the convolution block between encoder and decoder in U-Net. MSU-Net (73+concatenated) and MSU-Net (37+concatenated) are obtained after concatenated the convolution kernel with different receptive fields. MSU-Net (37+dilated) is obtained by dilated convolution. Intersection over Union (IoU) is used as the evaluation metric for comparison. Bold values represent the best results*.

**Figure 5:**
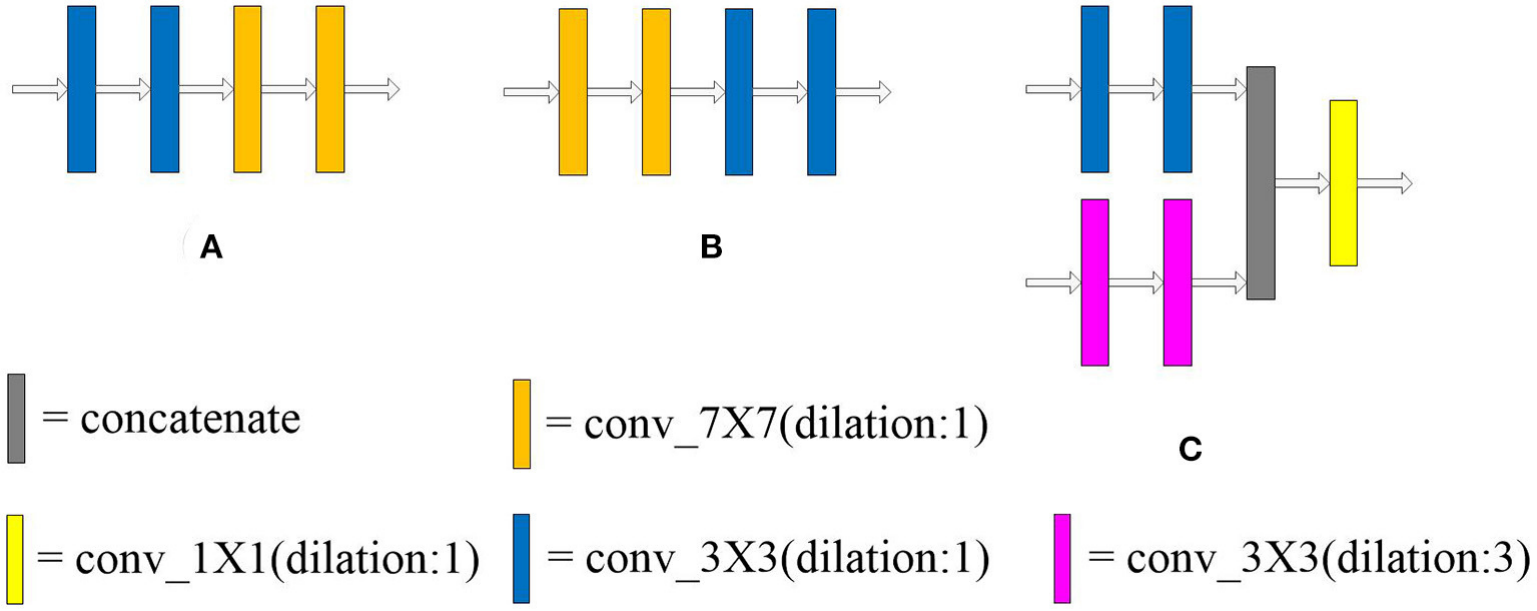
Arrangement of convolution kernel with different receptive fields and dilated convolution. Panels **(A,B)** lay out the convolution kernel in different order, respectively. In **(C)**, the large convolution kernel in the multi-scale block (37) is replaced by dilated convolution. The receptive field of the convolution kernel is enlarged without increasing the number of parameters.

The dilated convolution is introduced into the multi-scale block after the optimal convolution kernel is obtained. The dilated convolution used in the experiment is described in Figure 2. Convolution kernels with different receptive fields are concatenated to verify the effectiveness of the multiple convolution sequence. The details are shown in Figure 5. The experimental results are shown in Table 2.

### 3.2. Network Architecture

The architecture of MSU-Net is illustrated in Figure 1. MSU-Net has a contraction path and an expansion path. The network architecture follows encoder-decoder. In original U-Net, each block consists of two convolutional layers. However, there is still a drawback in this block. Due to the limitation of the receptive field, the network does not achieve better performance in feature extraction and feature restoration. The convolution blocks in encoder of the original U-Net are replaced with multi-scale blocks to obtain MSU-Net (encoder). The convolution blocks in decoder of the original U-Net are replaced with multi-scale blocks to obtain MSU-Net (decoder). The experimental results are illustrated in Table 2. In MSU-Net, the multi-scale block (37) is used to replace the all convolution block in the original U-Net. Multi-scale block enables encoder to extract more detailed information. Multi-scale block makes the features of decoder restoration more complete.

### 3.3. Extension of Model

**Figure 6:**
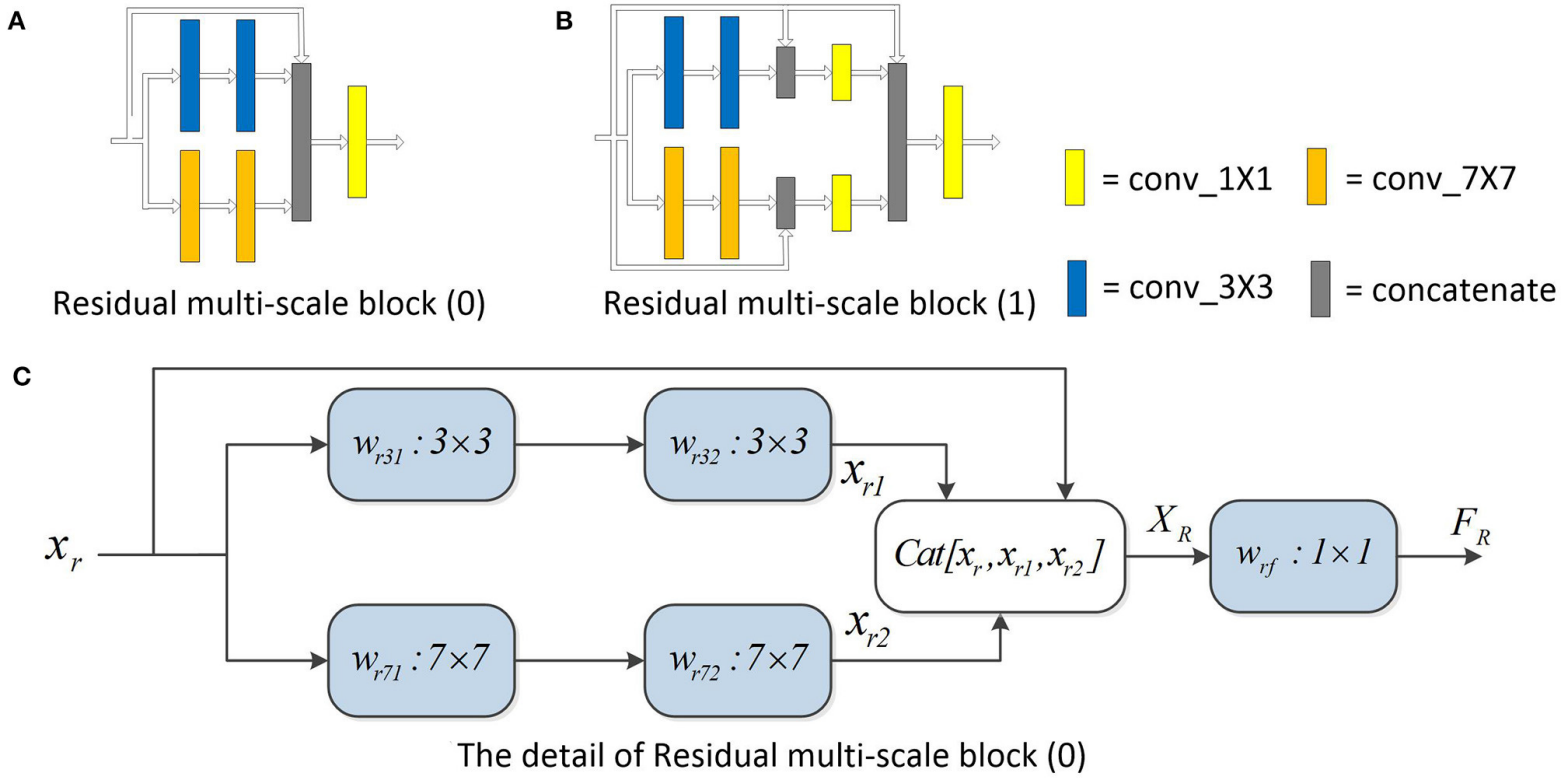
Residual multi-scale block. Panel **(A)** is the first structure designed to incorporate residual thinking. Panel **(B)** is the second. Experimental results show that the performance of **(A)** structure is better than **(B)**. Panel **(C)** describes **(A)** in detail. The different multi-scale blocks are described in Figure 3. The information of the input features is directly transmitted to the deep layer of the network through the residual connection.

Residual (He et al., 2016) is expanded into our model. The residual multi-scale block is shown in Figure 6. In addition, multi-scale blocks are also extended to variants of U-Net.

#### 3.3.1. Residual Multi-Scale Block

The idea of residual is introduced with multi-scale blocks to obtain residual multi-scale block (0) and residual multi-scale block (1). Residual multi-scale block (0) and residual multi-scale block (1) are shown in Figures 6A,B, respectively. The original convolution block in U-Net was replaced by residual multi-scale block (0) and residual multi-scale block (1) to get Res MSU-Net (0) and Res MSU-Net (1). The experimental results are described in **Table 4**. In **Table 4**, the performance of residual multi-scale block (0) is better than residual multi-scale block (1).

The structure of residual multi-scale block (1) is described below. *x*_*r*_ represents the characteristics of the input. *x*_*r*1_ and *x*_*r*2_ represent the characteristics obtained by the convolution kernel of different receptive fields. *F*_*R*_ is the output result of the multi-scale block. *F*_*R*_ is computed as follows:

(5) $$\begin{array}{l} x_{r1}=w_{r32}\left(w_{r31}x_{r}+b_{r31}\right)+b_{r32} \end{array}$$

(6) $$\begin{array}{l} x_{r2}=w_{r72}\left(w_{r71}x_{r}+b_{r71}\right)+b_{r72} \end{array}$$

(7) $$\begin{array}{l} X_{R}=Cat\left[x_{r},x_{r1},x_{r2}\right] \end{array}$$

(8) $$\begin{array}{l} F_{R}=w_{rf}X_{R}+b_{rf} \end{array}$$

Residual connection can make the forward and backward propagation of multi-scale block smoother. In forward propagation, the input signal can be propagated directly from the bottom to the top. The problem of network degradation can be alleviated. In back propagation, the error signal can be propagated directly to the lower layer without any intermediate weight matrix transformation. The problem of gradient dispersion can be alleviated. In addition, the generalization capacity of the network can be enhanced by the structure.

#### 3.3.2. Other Structures

In addition to combining the structure with our proposed multi-scale block, we also extend our multi-scale block on the variants of original U-Net. The convolution blocks in AttU-Net (Oktay et al., 2018) and U-Net++ (Zhou et al., 2020) are replaced with multi-scale block, namely MSAttU-Net and MSU-Net++, respectively.

## 4. Experiment

### 4.1. Dataset

Table 3 summarizes the five biomedical image segmentation datasets used in this study. These lesions/organs are derived from the most common medical imaging modalities, such as microscopy, X-ray, B-mode ultrasound, etc. The dataset was randomly divided into six subsets. Five of six are used as a training-validation dataset, and the remaining data as a test dataset. Five-fold cross validation is applied by randomly dividing training-validation into five subsets. The training process alternates with a fixed ratio of 4:1 between the training dataset and the validation dataset.

**Table 3:** Summary of biomedical image segmentation datasets used in our experiments.

**Applications**	**Images**	**Input size**	**Modality**	**Provider**
EM	30	512 × 512	Microscopy	ISBI 2012 (Cardona et al., 2010)
BUL	163	128 × 128	Ultrasound	Breast Ultrasound Lesions Dataset (Yap et al., 2017)
CXR	704	128 × 128	X-ray	Chest X-ray Database (Candemir et al., 2013; Jaeger et al., 2013)
SL	2594	256 × 256	Demoscopy	ISIC 2018 (Tschandl et al., 2018; Codella et al., 2019)
NS	30	512 × 512	Digitize	Kaggle

**Figure 7:**
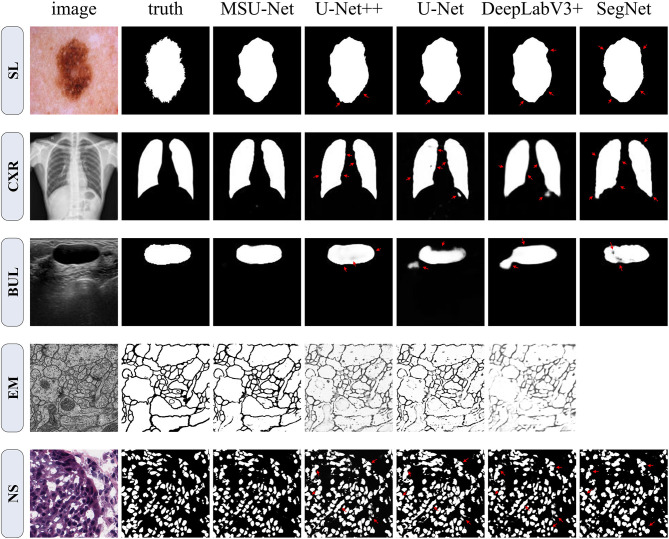
Qualitative comparison among SegNet, DeepLabV3+, U-Net, U-Net++, and MSU-Net. It shows the segmentation application of the architectures on five different biomedical image datasets. The red arrows indicate areas of incorrect segmentation. SegNet can not be trained on EM datasets. Therefore, the result of SegNet on the EM dataset is vacant. The ground truth is illustrated in the second column (from **left** to **right**).

*(1) Electron Microscopy (EM):* The dataset is provided by the EM segmentation challenge (Cardona et al., 2010), which is a part of ISBI 2012. The dataset contains 30 images (512 × 512 pixels) from a serial section Transmission Electron Microscopy (ssTEM) dataset of the Drosophila first instar larva ventral nerve cord (VNC). The images has not been resized. The images size of the input network is 512 × 512. An example of dataset is shown in Figure 7. Each image has a completely annotated ground truth segmentation map of the corresponding cell (white) and membranes (black).

*(2) Breast Ultrasound Lesions (BUL):* The Breast Ultrasound Dataset B (BUL) open-sourced in (Yap et al., 2017) is used in this study. This dataset includes 163 ultrasound images of breast lesions from different women. The image size of average is 760 × 570 pixels where each of the images presented one or more lesions. For our experiments, the data is resampled to 128 × 128 pixels. The ground truths provided in the BUL are in the form of binary masks of the lesions, as illustrated in Figure 7.

*(3) Chest X-ray (CXR):* The standard digital image database for Tuberculosis (Candemir et al., 2013; Jaeger et al., 2013) is created by the National Library of Medicine, Maryland, USA in collaboration with Shenzhen No.3 People's Hospital, Guangdong Medical College, Shenzhen, China. The Chest X-rays are from out-patient clinics. There are 800 images in the Chest X-rays dataset. However, the ground truth of 96 images is unknown. Seven hundred and four images of corresponding GT in the dataset were used by us. The image size of average is 4456 × 4456 pixels. The images are rescaled to 128 × 128 for this implementation. Referring to the example in Figure 7.

*(4) Skin Lesions (SL):* The dataset is provided by the ISIC 2018: Skin Lesion Analysis Toward Melanoma Detection grand challenge dataset (Tschandl et al., 2018; Codella et al., 2019). This dataset consists of 2594 RGB images of skin lesions with an average image size of 2166 × 3188 pixels. For our experiments, the dataset is resampled to 256 × 256 pixels with cross validation. The training samples include the original image and the binary image containing the lesion. Pixels outside the target lesion are represented by 0.

*(5) Nuclei Segmentation (NS):* This dataset is provided by The Cancer Genome Atlas (TCGA). This dataset can be downloaded from Kaggle. The dataset comprising 30 digitized Hematoxylin and Eosin (H&E)-stained frozen sections (512 × 512 pixels) derived from 10 different human organs. The dataset were selected from different laboratories to maximize the staining variability in the data set. Image tiles (3 per tissue) were extracted from adrenal gland, larynx, lymph nodes, mediastinum, pancreas, pleura, skin, testes, thymus, and thyroid gland. Like the EM dataset, this dataset was not sampled prior to input. The image size of the input is 512 × 512.

### 4.2. Baselines and Implementation

For comparison, the original U-Net is used to implement the segmentation task. U-Net is a common performance baseline for medical image segmentation. In addition, a wide U-Net with a similar number of parameters to our proposed architecture was designed. This is to ensure that the performance gain yielded by our architecture is not simply due to the increased number of parameters.

In this experiment, the program was based on the Pytorch (Paszke et al., 2019) framework. SGD (Robbins and Monro, 1951) was used as the optimizer with the learning rate of 1e-2. Both networks were constructed from the original U-Net. All the experiments are performed using an NVIDIA GeForce RTX 2080 Ti GPUs with 11 GB memory.

### 4.3. Evaluation Measures

In this paper, the Intersection over Union (IoU) is used as the main evaluation indicator to evaluate the results. Alternative measurement metrics could be found in **Table 6**, such as dice coefficient, precision, area Under Curve (AUC), and statistical analysis. These metrics were calculated as follows:

(9) $$\begin{array}{l} IoU=\frac{TP}{TP+FP+FN} \end{array}$$

(10) $$\begin{array}{l} Dice=\frac{2TP}{2TP+FP+FN} \end{array}$$

(11) $$\begin{array}{l} Precision=\frac{TP}{TP+FP} \end{array}$$

where TP, FP, and FN represent the number of true positive, false positive, and false negative, respectively. In addition, the area under receiver operation characteristic curve (AUC) is used to measure the segmentation performance. The closer the AUC is to 1.0, the higher authenticity of the segmentation method. When it is equal to 0.5, it has the lowest authenticity and no application value.

## 5. Results

### 5.1. Selection of Multi-Scale Block

31 kinds of multi-scale blocks were designed by combining the convolution kernel with different receptive fields. The different multi-scale blocks are shown in Figure 3. All multi-scale blocks were embedded into the original U-Net respectively. Subsequently, an ablation analysis of multi-scale block is made on three datasets. The experimental results of different multi-scale blocks on the dataset are illustrated in Table 1. Two key findings are illustrated in our results: (1) The wider network structure is not always better, (2) The optimal width of the network depends on the difficulty and size of the dataset. Although these findings may facilitate the automatic search of neural structures, this approach is hampered by limited computational resources (Elsken et al., 2018; Liu et al., 2018, 2019; Zoph et al., 2018).

The influence of the difference receptive field on the network performance is shown in Table 1. Among them, multi-scale block (37) achieves the best performance on datasets.

Different arrangements of convolution blocks and different convolution kernels are verified in Table 2. The robustness of the multiple convolution sequence is demonstrated by experimental results.

### 5.2. Results of the Extended Model

The multi-scale block was extended by us. First, the idea of residuals was introduced into the proposed module. Two multi-scale blocks based on residuals were constructed. The structure is shown in Figure 6. Second, the proposed multi-scale block was extended to the existing U-Net variants. Convolution kernel in AttU-Net and U-Net++ was replaced by multi-scale block. The experimental results are shown in Tables 4, 5. Experimental results show that the proposed method has good scalability and compatibility.

**Table 4:** Ablation study for U-Net, wide U-Net, MSU-Net, Res MSU-Net(0), and Res MSU-Net(1).

**Architecture**	**BUL**	**EM**	**NS**
	**M ± SD**	**M ± SD**	**M ± SD**
U-Net (Ronneberger et al., 2015)	0.608 ± 0.037	0.884 ± 0.007	0.675 ± 0.018
wide U-Net (Ours)	0.643 ± 0.025	0.889 ± 0.016	0.677 ± 0.012
MSU-Net (Ours)	0.708 ± 0.011	**0.900** **±** **0.001**	0.702 ± 0.010
Res MSU-Net (0) (Ours)	**0.713** **±** **0.032**	**0.900** **±** **0.001**	**0.704** **±** **0.010**
Res MSU-Net (1) (Ours)	0.628 ± 0.025	0.848 ± 0.056	0.675 ± 0.022

*Wide U-Net is obtained by extending the width of the U-Net network. The wide U-Net has the same number of parameters as the MSU-Net. Res MSU-Net (0)/Res MSU-Net (1) are proposed based on Residual multi-block. Intersection over Union (IoU) is used as the evaluation metric for comparison. Bold values represent the best results*.

**Table 5:** Ablation study for AttU-Net, MSAttU-Net, U-Net++, and MSU-Net++.

**Architecture**	**BUL**	**EM**	**NS**
	**M ± SD**	**M ± SD**	**M ± SD**
AttU-Net (Oktay et al., 2018)	0.607 ± 0.039	0.853 ± 0.043	0.655 ± 0.020
MSAttU-Net (Ours)	0.674 ± 0.005	0.895 ± 0.004	0.677 ± 0.010
U-Net++ (Zhou et al., 2020)	0.670 ± 0.020	0.885 ± 0.013	0.665 ± 0.012
MSU-Net++ (Ours)	0.687 ± 0.009	0.895 ± 0.002	0.691 ± 0.022

*MSAttU-Net and MSU-Net ++ are extended versions of AttU-Net and U-Net ++. Intersection over Union (IoU) is used as the evaluation metric for comparison*.

It can be seen from the experimental results that the performance of wide U-Net is better than U-Net. The main reason is that there are more parameters in wide U-Net. When the residual idea is not introduced, MSU-Net achieves very robust performance on all three data sets. Compared with U-Net, MSU-Net is higher than 0.1, 0.016, and 0.027 on the three datasets. The performance of the network is improved by introducing residual ideas. In addition, the extended experiment on U-Net variants also confirmed the effectiveness and universality of multi-scale block. By comparing the performance of MSU-Net (37+encoder) and U-Net, we found that the ability of network to extract features was enhanced by combining multi-scale blocks.

### 5.3. Semantic Segmentation Results

In order to verify the performance of the network, MSU-Net was compared with the current more advanced segmentation network (Ronneberger et al., 2015; Badrinarayanan et al., 2017; Chen et al., 2018b; Zhou et al., 2020). In addition, chest X-ray and skin lesion segmentation datasets were added to the experiment. These two datasets are larger than the three previously mentioned datasets. Figure 7 depicts a qualitative comparison of the results between the different split schemas. Compared with other architectures, the segmentation results of MSU-Net are more detailed. SegNet cannot be trained on EM datasets. Therefore, SegNet has not experimented on the EM dataset.

Table 6 shows the segmentation performance of the architectures on different datasets. A statistical analysis based on independent two-sample *t*-tests is performed by us for each pair of data between different structures. Our results show that MSU-Net is an effective network structure.

**Table 6:** Semantic segmentation results measured by different metrics for different network architectures.

**Metric**	**Architecture**	**SL**		**CXR**		**BUL**		**EM**		**NS**	
		**M ± SD**	**p-value**	**M ± SD**	**p-value**	**M ± SD**	**p-value**	**M ± SD**	**p-value**	**M ± SD**	**p-value**
IoU	SegNet (Badrinarayanan et al., 2017)	0.752 ± 0.007	9.824e-4	0.832 ± 0.008	6.179e-5	0.630 ± 0.033	0.001	—	—	0.586 ± 0.021	4.084e-6
	DeepLabV3+ (Chen et al., 2018b)	0.762 ± 0.002	2.202e-3	0.847 ± 0.005	3.261e-4	0.558 ± 0.034	1.761e-5	0.837 ± 0.015	1.582e-5	0.582 ± 0.019	1.717e-6
	U-Net (Ronneberger et al., 2015)	0.751 ± 0.005	1.872e-4	0.857 ± 0.005	0.020	0.608 ± 0.037	4.789e-4	0.884 ± 0.007	6.873e-4	0.675 ± 0.018	0.020
	U-Ne++ (Zhou et al., 2020)	0.746 ± 0.008	2.725e-4	0.863 ± 0.004	0.232	0.670 ± 0.020	0.013	0.885 ± 0.013	0.031	0.665 ± 0.012	8.243e-4
	MSU-Net(Ours)	**0.771** **±** **0.004**	—	**0.867** **±** **0.006**	—	**0.708** **±** **0.011**	—	**0.900** **±** **0.001**	—	**0.702** **±** **0.011**	—
Dice	SegNet (Badrinarayanan et al., 2017)	0.852 ± 0.006	0.002	0.908 ± 0.005	6.393e-5	0.770 ± 0.026	0.002	—	—	0.738 ± 0.017	5.941e-6
	DeepLabV3+ (Chen et al., 2018b)	0.857 ± 0.003	0.002	0.917 ± 0.003	3.123e-4	0.713 ± 0.029	3.215e-5	0.911 ± 0.009	2.104e-5	0.734 ± 0.016	2.830e-6
	U-Net (Ronneberger et al., 2015)	0.850 ± 0.004	1.696e-4	0.923 ± 0.003	0.020	0.753 ± 0.029	6.919e-4	0.938 ± 0.004	7.314e-4	0.805 ± 0.013	0.022
	U-Ne++ (Zhou et al., 2020)	0.847 ± 0.006	2.892e-4	0.926 ± 0.002	0.230	0.800 ± 0.014	0.015	0.939 ± 0.007	0.032	0.797 ± 0.008	5.129e-4
	MSU-Net(Ours)	**0.865** **±** **0.003**	—	**0.929** **±** **0.004**	—	**0.827** **±** **0.008**	—	**0.947** **±** **0.001**	—	**0.824** **±** **0.007**	—
Precision	SegNet (Badrinarayanan et al., 2017)	0.886 ± 0.010	0.161	0.856 ± 0.009	4.465e-4	0.725 ± 0.040	0.115	—	—	0.873 ± 0.008	0.203
	DeepLabV3+ (Chen et al., 2018b)	0.892 ± 0.008	0.037	0.875 ± 0.005	0.029	0.798 ± 0.054	2.227e-4	0.864 ± 0.029	6.076e-4	0.860 ± 0.019	0.065
	U-Net (Ronneberger et al., 2015)	**0.899** **±** **0.014**	0.024	0.878 ± 0.006	0.079	0.760 ± 0.061	0.018	0.913 ± 0.014	0.007	**0.888** **±** **0.019**	0.917
	U-Net++ (Zhou et al., 2020)	0.895 ± 0.010	0.030	0.882 ± 0.005	0.274	0.786 ± 0.043	0.011	0.919 ± 0.025	0.196	0.853 ± 0.059	0.267
	MSU-Net(Ours)	0.873 ± 0.015	—	**0.887** **±** **0.009**	—	**0.842** **±** **0.006**	—	**0.935** **±** **0.003**	—	0.887 ± 0.021	—

*We have performed independent two sample t-test between and highlighted boxes in red when the differences are statistically significant (p < 0.05). Bold values represent the best results*.

**Figure 8:**
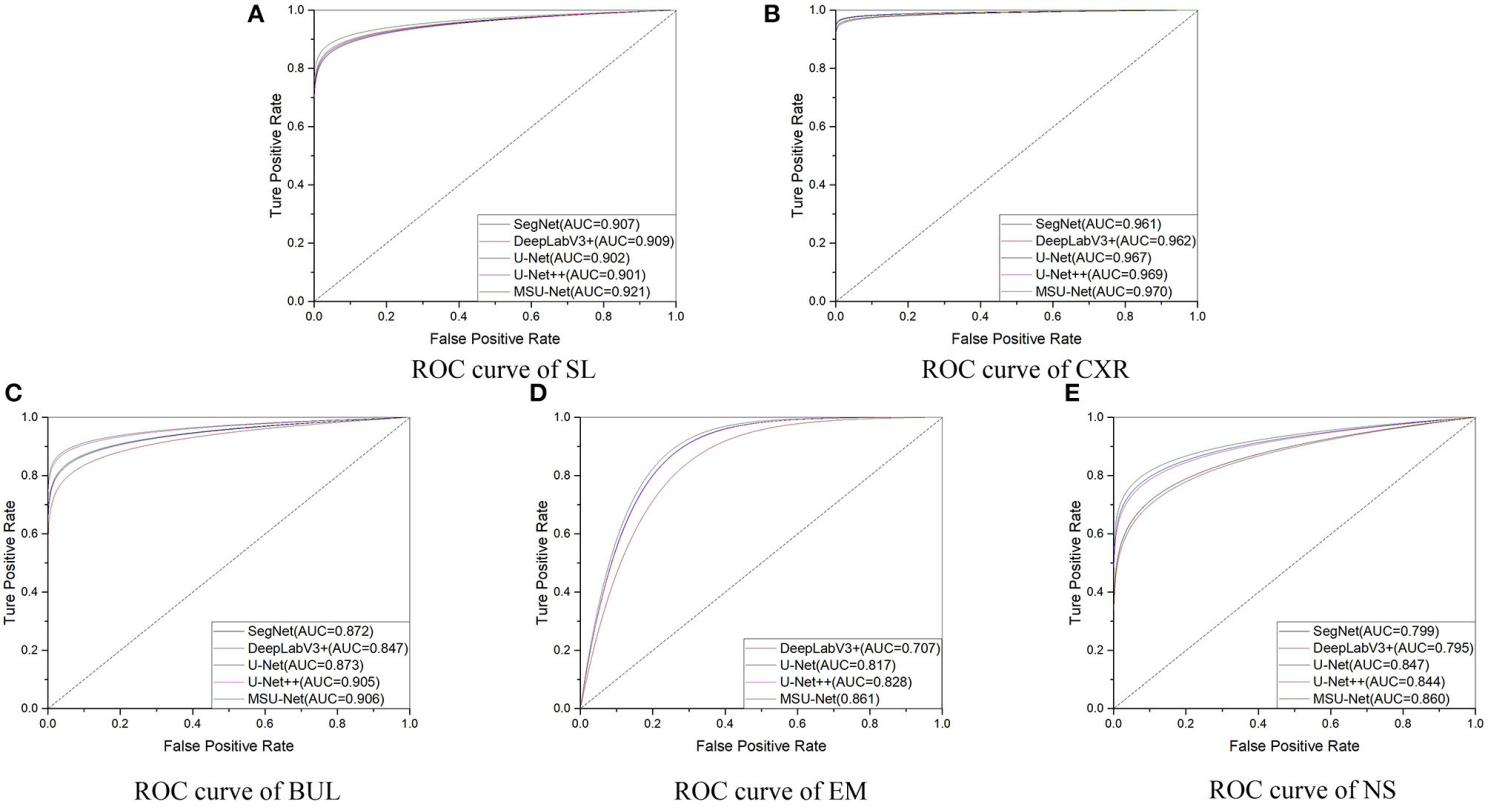
ROC curves for different architectures. AUC is the area under the curve.

The results in Table 5 suggest that our proposed MSU-Net is more robust in semantic segmentation. Compared with the U-Net, MSU-Net achieves a significant IoU gain over both architectures for all the five tasks of SL (↑0.01), CXR (↑0.01), BUL (↑0.1), EM (↑0.016), NS (↑0.027) segmentation. AUC of different architectures on the data set is illustrated in Figure 8. Figure 8 shows the ROC curve of different architectures on the datasets. Our model achieves the best performance in all datasets. Fine Precision is not captured by our model on the SL dataset. However, the high sensitivity of our model is shown in Figure 8. This allows false positives and false negatives in the data to be better balanced by our model. It is mainly due to the multiple convolution sequence with different receptive fields. This design makes the features in the network richer and more diverse.

## 6. Discussion

Medical image segmentation plays an important role in diagnosis, treatment and prognosis evaluation. In the process of diagnosis, the main applications include morphological analysis, volume calculation, anatomical structure analysis, etc. In surgical treatment planning, the commonly used methods include preoperative biopsy guidance, target area planning of radiotherapy, image registration fusion and path planning, and target tracking in medical robot, etc. In the prognostic assessment, the most important segmentation is the analysis of lesion volume change and the analysis of lesion histological characteristics. In addition, medical image segmentation can be applied to three-dimensional reconstruction visualization, which can provide clinicians with more intuitive pathological morphology and spatial anatomy. In recent years, the method based on deep learning has been widely used in medical image segmentation. However, the performance of segmentation is greatly affected by the network architecture and the ability to acquire features in learning process.

U-Net is a very classical network architecture in the field of medical image segmentation. At present, U-Net is widely used in medical image segmentation. However, the basic architecture of U-Net has not been significantly modified by the researchers. Large receptive fields play an important role when we need to make dense per-pixel predictions. In order to improve the existing segmentation model, multi-scale blocks are constructed by convolution sequence and multiple convolution kernel with different receptive fields. The different types of multi-scale blocks are illustrated in Figure 3. In addition, MSU-Net is proposed after all the convolution blocks in the original U-Net are replaced by multi-scale block. The details of the MSU-Net are illustrated in Figure 1. Multiple convolution sequences are used to extract more semantic features from images. In addition, convolution kernels with different receptive fields are used to make features more diverse. The problem of unknown network width is alleviated by effective integration of multiple convolution sequences with different receptive fields.

The most important innovation described in this paper is the combination of multiple convolution sequences and convolution kernel with different receptive fields to improve the segmentation performance. It can be seen from the Table 1 that the performance of the network is affected by different receptive fields. Good performance was achieved by combining advanced ideas with multi-scale blocks. In addition, multi-scale blocks are extended to the variants of original U-Net. The results in Tables 4, 5 describes that the segmentation performance of the network is improved by combining the multiple convolution sequence and the convolution kernel with the different receptive fields. The strategies of our proposed strategy has the following advantages: (1) More diverse features are extracted through the convolution kernel of different receptive fields. This is useful for intensive forecasting tasks that require detailed spatial information. At the same time, the problem of unknown network width can be alleviated. (2) More feature information is extracted by multi-convolution sequence, which is helpful to the segmentation task. Our method has obtained the best performance compared with the advanced models through the demonstration of multiple medical image segmentation datasets (see in Table 6). The highest AUC is obtained by our architecture (see in Figure 8). This suggests that our model has a stronger ability to balance false positives and false negatives in the data. In general, the proposed method is useful for intensive forecasting tasks requiring detailed spatial information. Different receptive fields can provide diverse semantic information for tasks, which is beneficial to the segmentation of lesions. More detailed segmentation results can provide doctors with more detailed lesion areas, which is helpful for the diagnosis of disease and the formulation of treatment plan.

Although we have widely evaluated the performance of the network on different datasets, there are still some deficiencies in our network. First, the convolution kernels with a larger receptive field are not attempted due to objective factors. The performance of the network may be improved through greater receptive field. Second, the dilated convolution can increase the receptive field of the convolution kernel without increasing the number of parameters. Unfortunately, dilated convolution was not attempted in our experiment. Third, our network has not been validated against the 3D medical image segmentation dataset. The above work may be completed by us in the future.

## 7. Conclusion

In order to obtain more accurate segmentation image, a new structure called multi-scale block was proposed by us. The convolution blocks in the original U-Net are replaced by multi-scale blocks to obtain MSU-Net. The improvement of MSU-Net performance is attributed to multiple convolution sequence and convolution kernels with different receptive fields. Two key issues are addressed by this design: (1) The diversity of features is lost due to the fixed size of the convolution kernel. (2) Feature information may be lost at each scale using a single convolutional sequence to extract features. Five different public datasets were used to conduct an extensive evaluation of MSU-Net. The experimental results show that MSU-Net achieves the best performance.

## Data Availability Statement

Publicly available datasets were analyzed in this study. This data can be found here: all datasets can be found in Table 3.

## Author Contributions

RS, DZ, and JL: conceptualization and writing (review and editing). RS, DZ, and CC: data curation. RS and DZ: methodology, validation, and writing (original draft). RS: project administration and visualization. All authors have read and agreed to the published version of the manuscript.

## Conflict of Interest

The authors declare that the research was conducted in the absence of any commercial or financial relationships that could be construed as a potential conflict of interest.
